# Perspectives on sipuleucel-T: Its role in the prostate cancer treatment paradigm 

**DOI:** 10.1080/2162402X.2015.1107698

**Published:** 2015-12-10

**Authors:** James L. Gulley, Peter Mulders, Peter Albers, Jacques Banchereau, Michel Bolla, Klaus Pantel, Thomas Powles

**Affiliations:** a Genitourinary Malignancies Branch and Laboratory of Tumor Immunology and Biology, National Cancer Institute, National Institutes of Health, Bethesda, MD, USA; b Radboud University Nijmegen Medical Center, Nijmegen, The Netherlands; c Düsseldorf University, Medical Faculty, Düsseldorf, Germany; d Jackson Laboratory for Genomic Medicine, Farmington, CT, USA; e Department of Radiation Therapy, C.H.U. Grenoble, Grenoble, France; f Department of Tumor Biology, University Medical Center Hamburg Eppendorf, Hamburg, Germany; g Barts Cancer Institute, Queen Mary University of London, London, UK

**Keywords:** Clinical trials, immune responses, immunotherapy, prostate cancer, sipuleucel-T

## Abstract

Sipuleucel-T is an autologous cellular immunotherapy approved in the US for patients with asymptomatic or minimally symptomatic metastatic castration-resistant prostate cancer (mCRPC). This significant advance for mCRPC treatment provides healthcare professionals with another effective therapy to extend survival. As an immunotherapy, sipuleucel-T possesses specific characteristics differentiating it from traditional therapies. At a roundtable meeting of experts, sipuleucel-T data were discussed, focusing on interpretation and clinical implications. Important differences between immunotherapies and traditional therapies were explored, e.g., mode of action, outcomes, data consistency and robustness, timing of sipuleucel-T treatment, and future perspectives in areas such as short-term markers of long-term benefit.

## Abbreviations

AE: adverse event
APC: antigen-presenting cell
CD: cluster of differentiation
CI: confidence interval
ECOG, Eastern Cooperative Oncology Group; FDA: Food and Drug Administration
GM-CSF: granulocyte-macrophage colony stimulating factor
HR: hazard ratio
IFNγ: interferon gamma
Ig: immunoglobulin
mCRPC: metastatic castration-resistant prostate cancer
NIH: National Institutes of Health
OS: overall survival
PAP: prostatic acid phosphatase
PBMC: peripheral blood mononuclear cell
PSA: prostate-specific antigen
SD: standard deviation
TNC: total nucleated cell
UCSF: University of California, San Francisco

## Introduction

Sipuleucel-T is an autologous cellular immunotherapy, the first approved by the United States Food and Drug Administration (FDA), and indicated for the treatment of asymptomatic or minimally symptomatic mCRPC.^1^ This article aims to provide a timely summary of sipuleucel-T clinical development, key data and current thinking on the implications of those data. We also explore the important differences between immunotherapies and traditional therapies in terms of mode of action and patient outcomes.

## Methods

Sipuleucel-T data were discussed by the authors at a roundtable meeting. This article is based on a discussion and interpretation of the available data. The authors focused specifically on information that could have important implications for clinical practice or for the validity of clinical trial data, and discussed the clinical relevance and applicability of the available evidence.

## Findings

### Overview of sipuleucel-T clinical development

Sipuleucel-T is manufactured by culturing the patient's own purified peripheral blood mononuclear cells (PBMCs) with PA2024. PA2024 is a fusion protein of prostatic acid phosphatase (PAP) and granulocyte-macrophage colony stimulating factor (GM-CSF) that promotes the differentiation of mononuclear cells, such as monocytes, into dendritic cells (a type of antigen-presenting cell; APC).^1,2^ The resulting cell product (Table 1)^3^ is then reinfused back into the patient, where the cells generate PA2024- and PAP-specific immune responses.^4^ Sipuleucel-T treatment involves three cycles of leukapheresis, *ex vivo* culture and reinfusion at 2-week intervals, resulting in an approximately 4-week treatment course.^1^

**Table 1.** Phenotype of cells within the sipuleucel-T product from Phase-2 clinical trials.

Number of products	66
Nucleated cells × 10^6^, median (range)	2,376 (216–3,108)
CD54^+^ cells (presumed dendritic cells) × 10^6^, median (range)	278 (18.6–1,276)
Proportion of cells positive for phenotype markers, mean ± SD	
CD54^+^ (dendritic cells)	14.8 ± 12.3
CD3 (T cells)	58.5 ± 15.5
CD19 (B cells)	6.7 ± 2.8
CD14 (monocytic cells)	14.8 ± 11.1
CD56 (natural killer cells)	14.6 ± 6.7

Abbreviation: SD, standard deviation. From Small EJ, et al. J Clin Oncol 2000;18(23):3894-903.3 Reprinted with permission. © 2000 American Society of Clinical Oncology. All rights reserved.

**Figure 1.**
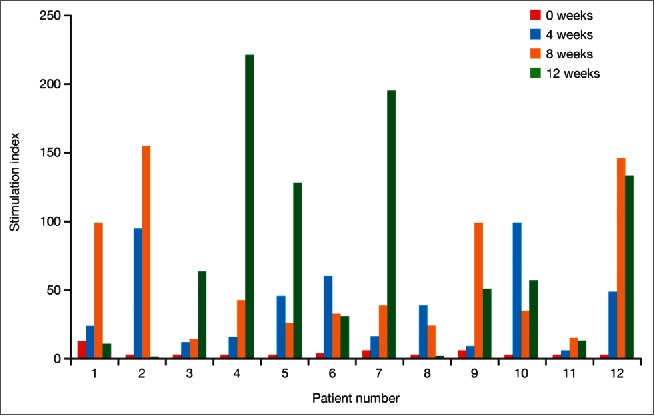
T-cell proliferation responses to the sipuleucel-T fusion protein (PA2024) in individual patients from Phase-1 studies. Standard T-cell proliferation assays were conducted and data are reported as the stimulation index (mean counts per minute with PA2024 / mean counts per minute with control). From Small EJ, et al. J Clin Oncol 2000;18(23):3894-903.3 Reprinted with permission. © 2000 American Society of Clinical Oncology. All rights reserved.

The concept of sipuleucel-T treatment originated from studies in lymphoma, where antigen-loaded, autologous APCs showed clinical promise.^5^ PAP was selected as an appropriate target antigen in prostate cancer as it is highly expressed in, and has a high degree of specificity for, prostate cells.^6^ Following preclinical success, Phase-1 and -2 clinical trials of sipuleucel-T at the Mayo Clinic and the University of California, San Francisco (UCSF) found that treatment was generally well tolerated, with no dose-limiting toxicities.^3^ In these Phase-1 studies, maximum immune responses to PA2024 (assessed by T-cell proliferation) were reached for individual patients after either two or three infusions (Fig. 1).^3^ The overall response was significantly higher at week 4 versus week 0 (*p* < 0.01) and at week 8 versus week 4 (*p* < 0.05), but not at week 12 versus week 8.^3^ Immune responses generated by sipuleucel-T treatment were specific to PA2024; responses to a recall antigen, influenza, were measured before and every four weeks during treatment and did not change.^3^

Although it became available relatively recently, sipuleucel-T clinical trials were initiated in the ‘docetaxel era’ using endpoints that were developed for new therapies at the time. The first sipuleucel-T Phase-3 trials (D9901 and D9902A) used the traditional measure of response, time to disease progression, as the primary endpoint.^7,8^ This endpoint was not met, but there was a significant benefit in the pre-specified endpoint of 3-year survival with sipuleucel-T versus placebo in D9901 (median survival benefit 4.5 months; *p* = 0.01; hazard ratio [HR] 0.586; 95% confidence interval [CI] 0.39–0.88).^1,7^ The subsequent IMPACT trial (D9902B; NCT00065442) met its primary endpoint of significantly improved overall survival (OS) with sipuleucel-T versus placebo (median survival benefit 4.1 months; *p* = 0.03; HR 0.78; 95% CI 0.61–0.98).^9^ Overall, in an integrated analysis of survival across the three trials (D9901, D9902A, and IMPACT; *n* = 737), sipuleucel-T provided a survival benefit compared with placebo (*p* < 0.001; HR 0.735 [95% CI 0.613–0.882]).^10^

As would be expected given the immunological mechanism of action of sipuleucel-T, significant associations were observed between OS and various immune parameters measured during treatment. There were significant correlations between OS and the cumulative total nucleated cell (TNC) count, APC count (cluster of differentiation [CD]54^+^ cells), and APC activation (CD54^+^ upregulation) measured during *ex vivo* culture of the patients' cells across all three cycles of treatment (Table 2).^4^ *In vitro* APC activation and measurable antigen-specific immune responses were observed during the second and third sipuleucel-T treatment cycles,^4^ consistent with an immunological ‘prime-boost’ effect. Adaptive immune responses against PA2024 could also be detected in patients following treatment with sipuleucel-T, and also correlated with OS (Table 2).^4^ This included functional T-cell responses (proliferation and interferon gamma [IFNγ] production) and antibody responses.

**Table 2.** Overall survival according to sipuleucel-T product characteristics and antigen-specific immune responses.^4^

	HR	95% CI	*p* value	Adjusted *p* value
APC activation > 26.69 (*n* = 238)	0.76	0.58 – 0.99	0.002	0.041
APC activation ≤ 26.69 (*n* = 238)				
APC count > 1.84×10E9 (*n* = 238)	0.79	0.68–0.93	0.016	0.005
APC count ≤ 1.84×10E9 (*n* = 238)				
TNC count > 9.7×10E9 (*n* = 238)	0.71	0.59–0.87	< 0.001	< 0.001
TNC count ≤ 9.7×10E9 (*n* = 238)				
PA2024 and/or PAP response (*n* = 123)	0.47	0.29–0.78	< 0.001	0.003
No response (*n* = 33)				
PA2024 response (*n* = 122)	0.46	0.28–0.76	< 0.001	0.002
No response (*n* = 34)				
PAP response (*n* = 60)	0.53	0.31–0.90	0.029	0.019
No response (*n* = 92)				

Abbreviations: APC, antigen-presenting cell; PAP, prostatic acid phosphatase; TNC, total nucleated cell. *p* values are from analyses with and without adjustment for baseline PSA and LDH.

Importantly, the immune responses generated by sipuleucel-T are long-lasting. Antigen-specific adaptive immune responses could be detected in the blood for at least 26 weeks after treatment in the majority of patients in pooled data from the IMPACT, D9901, and D9902A studies.^4^ In the prospective, randomized Phase-3 PROTECT trial (NCT00779402), a small number of patients with androgen-dependent prostate cancer progressed to mCRPC and were therefore eligible for sipuleucel-T retreatment in the open-label P10–1 study (NCT01338012). Antigen-specific IFNγ responses were present at baseline in these patients and retreatment took place a median of 8.6 years after first sipuleucel-T treatment.^11^ Immunoglobulin class switching, or isotype switching, (immunoglobulin [Ig] M to IgG antibodies) was also demonstrated following treatment.^4^ Interestingly, a transient increase in peripheral blood eosinophil counts was observed in the IMPACT study from baseline to week 6 and correlated with OS,^12^ although the clinical significance of this remains unclear. Preliminary evidence also suggests that peripheral blood immune responses translate to effects within the prostate; a statistically significant ≥ 3-fold increase in T cells was observed at the tumor interface compared with pretreatment biopsies or with non-interface areas of surrounding benign or malignant tissue (*p* < 0.001 for each comparison).^13^

Sipuleucel-T also has a favorable and generally manageable adverse event (AE) profile. In clinical trials, AEs were mainly mild or moderate and infusion-related with a low discontinuation rate.^1,8,9^ The majority of AEs were acute infusion reactions, which mostly resolved within 48 h.^1,8,9^ Some cerebrovascular events were noted,^1,9^ and will be further explored in the PROCEED study (NCT01306890).

### Treatment endpoints and response kinetics

**Figure 2.**
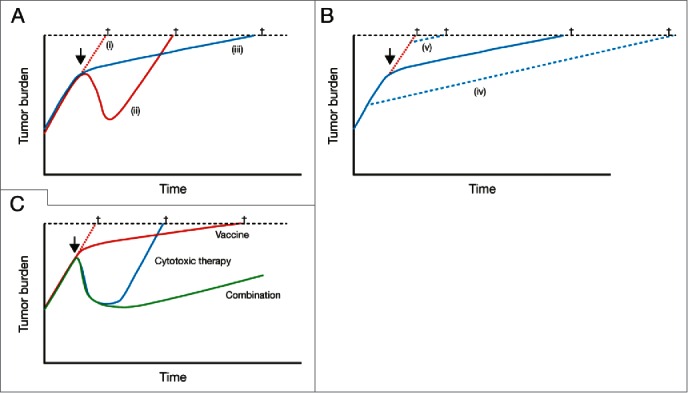
Model reconciling the lack of short-term effects of immunotherapy with the long-term OS benefit versus placebo. (A) Comparison of disease kinetics with (i) no treatment, (ii) traditional cytotoxic therapy, or (iii) immunotherapy. (B) Initiation of immunotherapy (iv) early in the disease course versus (v) in patients with late-stage disease. (C) Combinations of cytotoxic therapy with immunotherapies could be potentially useful future treatment options, combining rapid tumor cell death with a long-term benefit related to the induction of immune responses. The arrows indicates the initiation of treatment, the crosses indicate cancer-related death. From Schlom J. J Natl Cancer Inst 2012;104(8):599-613,17 by permission of Oxford University Press.

In terms of survival, sipuleucel-T treatment has a significant effect on OS but a lack of effect on earlier endpoints such as time to disease progression.^7-9^ Overall, and in contrast to chemotherapy or hormonal therapies, the mechanism of action of immunotherapies appears to alter the long-term course of the disease and demonstrates a delay in response.^14-17^ This appears to be a class effect, which is exemplified by a divergence in OS that is seen at approximately 6 months after treatment with sipuleucel-T versus control.^9^ Rather than providing the rapid tumor cell apoptosis or death associated with the use of traditional therapies, immunotherapies may slow or stop the accumulation of tumor cells via a dynamic immune response (Fig. 2A).^16-19^ Indeed, prostate-specific antigen (PSA) doubling time was significantly lengthened with sipuleucel-T versus control in patients with rising PSA after surgery (48% increase in PSA doubling time; *p* = 0.038).^20^ This sustained slowing of tumor growth rate seen with immunotherapies suggests that using them early in the mCRPC therapeutic landscape may lead to improved outcomes, compared with use later in the disease.

In terms of other endpoints, the benefit of sipuleucel-T over placebo was greater with long- and medium-term versus short-term outcomes. Sipuleucel-T treatment produced a significant benefit over placebo in OS (long-term) and time to first use of an opioid analgesic for cancer pain (medium-term), without a significant effect on shorter-term outcomes such as disease-related progression.^9,21^ In this regard, short-term markers of future benefit from sipuleucel-T, or other immunotherapies, are not currently available in the clinic and would be very useful. In fact, the transient increase in eosinophil counts noted in many patients' peripheral blood between baseline to week 6 following sipuleucel-T treatment correlated with OS.^12^ This observation may be clinically useful once validated, as eosinophil counts can be routinely measured.

Considering these characteristics of sipuleucel-T treatment, it is important that healthcare providers explain to patients what they can expect from this therapy. The majority of mCRPC patients are eligible to receive sipuleucel-T, and the short treatment course may be considered favorable. The idea that the patients' bodies are working to fight the cancer can also be appealing. However, it is important that patients understand the likely outcome of sipuleucel-T treatment, as this differs from other therapies that they may have received. Therefore, it is essential that healthcare provider teams effectively communicate how sipuleucel-T works, explaining that patients may not see changes in traditional markers of treatment success such as PSA levels.

Overall, despite the delay in response, the long-term immunological memory generated by sipuleucel-T treatment could enable the body to continue to fight tumor cells and slow their accumulation and spread long after immunotherapy has been completed, which may help to explain the long-term survival benefit of sipuleucel-T. Furthermore, theoretically, long-term, systemic immunosurveillance could act throughout the body as well as within the prostate and could, therefore, potentially target metastatic cells before they become established.

### Robustness of sipuleucel-T efficacy data

#### Treatment effects across patient subgroups

Of over 49 individual subgroups assessed for the IMPACT trial, the OS treatment effect favored sipuleucel-T over placebo in all but the subgroup of patients aged <65 years.^9,22^ This analysis appears to be a statistical type-1 error (false positive). There was a positive effect above and below the median age of 71 years in IMPACT,^9^ and the discrepancy between age groups was not seen consistently across studies. An independent FDA reviewer conducted an integrated analysis across all three Phase-3 studies and a separate analysis in study D9901, both of which showed a benefit in patients aged <65 years.^22^ The FDA concluded that this supported the hypothesis that the subgroup of subjects aged <65 years also benefit from treatment with sipuleucel-T and that the finding in IMPACT most likely resulted from chance, related to the multiplicity of comparisons in 49 different subgroups.^22^

#### Appropriateness of control groups

In IMPACT, sipuleucel-T was generated using all or most of the cells obtained by leukapheresis. The control group underwent a sham procedure whereby they received a placebo infusion prepared by culturing one-third of the cells collected by leukapheresis (as per the sipuleucel-T arm) at 2–8°C and without PA2024.^9^ The remaining cells were preserved for potential future use in a salvage study. Also, it has been speculated that removal of large proportions of circulating lymphocytes by leukapheresis may negatively impact some patients and their immune systems. However, there is no evidence to suggest that immunodepletion occurred for patients in the control arm in IMPACT. The number of cells removed by leukapheresis represents approximately 0.1–1.4% of the total body pool of lymphocytes.^23^ Lymphocyte populations rapidly regenerate and equilibrate,^24,25^ and the median lymphocyte and monocyte counts remained within the normal range throughout treatment.^26^ Furthermore, no increase in infections was seen in the control arm (27.7% of patients) relative to the sipuleucel-T arm (27.5% of patients).^27^ Indeed, multiple studies of repeated apheresis procedures on healthy donors have shown no detrimental effects.^28-33^

These clinical trial data suggesting that leukapheresis did not selectively compromise control patients in IMPACT are also supported by experience from the United States National Institutes of Health (NIH).^29,34^ In this analysis, 4,957 serial donations were obtained from more than 400 individuals between January 1995 and December 2001, with a median 6.8 L per procedure. The majority of patients had a subsequent leukapheresis within 56 d of the previous procedure (*n* = 3,370, median 23 d), and the median change in absolute lymphocyte count was −3.6%. After 2–9 procedures, the median lymphocyte count had decreased by < 10%, which was considered to be clinically insignificant. No increased susceptibility to infectious diseases or cancer was observed.^29^

#### Impact of post-randomization therapies

Docetaxel was the only therapy with demonstrated OS benefit that was commercially available for use during the follow-up period of the pivotal sipuleucel-T IMPACT study. The use of docetaxel after sipuleucel-T, therefore, has the potential to affect OS and it should be noted that docetaxel is given with prednisone, which may be immunosuppressive. Importantly, a sipuleucel-T treatment effect was observed both in patients who did (HR 0.825; 95% CI 0.619–1.101) and did not (HR 0.693; 95% CI 0.545–0.880) receive subsequent docetaxel, and the overall sipuleucel-T treatment effect remained robust when adjusting for docetaxel use.^9,10^ An exploratory analysis also suggested that the timing of docetaxel initiation was unlikely to have affected the significant OS benefit with sipuleucel-T.^22^ Other therapies reported as being used were other chemotherapy and hormone therapy (excluding medical castration);^22^ potential exposure to cabazitaxel, Ra-223, enzalutamide, and abiraterone acetate/prednisone was limited as they were only available in clinical trials at this time.

#### Treatment sequencing and combinations

Since 2010, several new therapies have become available to treat patients with mCRPC. Clinical trials have evaluated these therapies and have shown both abiraterone acetate and enzalutamide to have efficacy in treating patients who had received prior docetaxel as well as those patients who had not previously been treated with docetaxel,^35-38^ cabazitaxel in patients who had received prior docetaxel therapy^39^ and radium-223 post-docetaxel treatment or in patients who could not tolerate or refused docetaxel.^40^ With these new therapies entering the treatment landscape in mCRPC, questions regarding therapy sequencing and combinations are increasingly important, and studies are on-going to better understand the position of sipuleucel-T within the prostate cancer treatment paradigm. Emerging data suggest a greater magnitude of benefit with earlier use of sipuleucel-T in patients with lower disease burden. In addition, preliminary data are becoming available on the use of sipuleucel-T before, or concurrently with, other therapies.

#### Treating patients with lower disease burden

From a theoretical perspective, it seems rational that early treatment with immunotherapy may provide the maximum benefit to patients.^15,41^ The current body of evidence indicates that immunotherapy has relatively slow response kinetics, and therefore treating patients early may allow them more time to benefit from the survival advantage provided by sipuleucel-T treatment (Fig. 2B).^17^ In addition, patients may have a more responsive immune system earlier in the disease course, when the number of immune-depleting therapies that have been used is more limited. For example, a significantly greater magnitude of *in vitro* APC activation (measured by cumulative CD54^+^ upregulation) during the sipuleucel-T manufacturing process was observed in clinical trials enrolling patient populations with earlier-versus-later-stage disease (neoadjuvant setting versus mCRPC; *p* < 0.001).^42^ Clinical trials showed a correlation between cumulative APC activation during sipuleucel-T treatment and OS.^4^

These concepts are supported by analyses from the IMPACT trial in which the greatest magnitude of benefit with sipuleucel-T was observed among patients with better baseline prognostic factors, particularly among patients with lower baseline PSA values.^9,43^ This supports the concept that patients with less advanced disease or lower disease burden may benefit the most from sipuleucel-T treatment and is in contrast to treatment with docetaxel, abiraterone, and enzalutamide, where a trend toward a greater OS benefit was observed with PSA above rather than below the median.^35,38,44^

Overall, these considerations provide a rationale for including immunotherapy as an early treatment strategy in treatment algorithms for patients with a lower burden of disease.

#### Treatment sequencing and combinations

The ability to treat the patient with other agents before, during and after sipuleucel-T could have an important impact on its optimal position within the prostate cancer treatment paradigm and may have wider implications for immunotherapy in general (Fig. 2C). STAMP is investigating the combination of sipuleucel-T and abiraterone acetate plus prednisone (concurrent treatment arm), or sipuleucel-T followed by abiraterone acetate plus prednisone (sequential treatment arm). Data suggest that immune parameters, such as cumulative APC activation, antibody responses and T-cell responses, were not affected by the concurrent use of abiraterone acetate and prednisone with sipuleucel-T (all *p* > 0.05 between treatment arms).^45^ This combination also appeared to be reasonably well tolerated, with similar side effect profiles between concurrent and sequential administration.^45^ An ongoing study will also investigate the concurrent or sequential use of sipuleucel-T and enzalutamide (P12–2; NCT01981122).

The AE profile of sipuleucel-T may offer advantages to physicians when considering potential treatment combinations and sequences. Sipuleucel-T AEs were mainly infusion- related^8,9^ and did not overlap with those typical of chemotherapy or androgen-deprivation therapy. This further supports the potential use of sipuleucel-T concomitantly or sequentially with other therapies.

Finally, when considering long-term treatment algorithms, the potential for retreatment with an effective agent should be considered. In the PROTECT trial (NCT00779402), patients treated with sipuleucel-T for androgen-dependent prostate cancer could receive an optional, single booster infusion of sipuleucel-T after biochemical failure.^20^ After boosting, sipuleucel-T-induced immune responses were maintained and were increased in some patients. Subsequent to this, as mentioned above, a small number of patients from the PROTECT trial received a second course of sipuleucel-T a median of 8.6 years later in the P10–1 study.^11^ Not only were long-term immune responses detected prior to retreatment with sipuleucel-T, these were also boosted after just one sipuleucel-T treatment.^11^ Therefore, for patients who receive sipuleucel-T at a relatively early disease stage, there is the potential for booster treatments to maintain this benefit in the long term.

The National Comprehensive Cancer Network (NCCN) has developed evidence-based guidelines for the treatment of a number of neoplasms. In 2015, the NCCN updated their prostate cancer treatment guidelines. These guidelines place immunotherapy with sipuleucel-T as a category-1 first line therapy in asymptomatic or minimally symptomatic patients who do not have liver metastases, have a life expectancy greater than six months and Eastern Cooperative Oncology Group(ECOG) performance status of 0–1. Following sipuleucel-T, the NCCN guidelines list enzalutamide, abiraterone acetate, docetaxel, and radium-223 as category-1 options for patients with no visceral metastases and docetaxel and enzalutamide as category-1 options for patients with visceral metastases.^46^ By placing sipuleucel-T as the first-line treatment in appropriate patients, these patients may receive immunotherapy when their tumor burden is lower, potentially maximizing the benefit of this treatment.

## Conclusions

Sipuleucel-T represents a significant advance in the treatment of mCRPC, providing uro-oncologists with an additional effective therapy to extend survival. Immunotherapies, such as sipuleucel-T, have specific characteristics that differ from traditional therapies. Sipuleucel-T generates a long-lived immune response and achieves a significant survival benefit for patients with mCRPC. The benefit of sipuleucel-T versus placebo in clinical trials is strongly supported by data showing an overall treatment effect regardless of age, and robust control data that do not support the idea that this patient group was selectively immunocompromised by leukapheresis. There is a growing body of evidence to suggest a greater magnitude of benefit with earlier use of sipuleucel-T in patients with lower disease burden. In this context, future research into the quantification and characterization of circulating tumor cells^47-50^ might complement established markers for estimating disease burden and assessment of therapy responses. Preliminary data on sequential or concurrent use with other therapies, including abiraterone acetate, are also encouraging. Most AEs with sipuleucel-T were infusion-related, and the safety profile does not overlap with that of other therapies for prostate cancer.
